# Microfluidic Detection of SPIONs and Co-Ferrite Ferrofluid Using Amorphous Wire Magneto-Impedance Sensor

**DOI:** 10.3390/s24154902

**Published:** 2024-07-28

**Authors:** Gabriele Barrera, Federica Celegato, Marta Vassallo, Daniele Martella, Marco Coïsson, Elena S. Olivetti, Luca Martino, Hüseyin Sözeri, Alessandra Manzin, Paola Tiberto

**Affiliations:** 1 Department of Advanced Materials Metrology and Life Science, Istituto Nazionale di Ricerca Metrologica (INRiM), Strada delle Cacce, 91, 10135 Turin, Italy; f.celegato@inrim.it (F.C.); marta.vassallo95@gmail.com (M.V.); m.coisson@inrim.it (M.C.); e.olivetti@inrim.it (E.S.O.); l.martino@inrim.it (L.M.); a.manzin@inrim.it (A.M.); p.tiberto@inrim.it (P.T.); 2 European Laboratory for Non Linear Spectroscopy (LENS), via N. Carrara, 1, 50019 Florence, Italy; martella@lens.unifi.it; 3 Department of Chemistry “Ugo Schiff”, University of Florence, Via della Lastruccia 3-13, 50019 Florence, Italy; 4 Magnetics Laboratory, TÜBITAK Ulusal Metroloji Enstitüsü (UME), Gebze Yerleşkesi, 41470 Kocaeli, Turkey; huseyin.sozeri@tubitak.gov.tr

**Keywords:** magneto-impedance sensor, magnetic nanoparticles, magnetic wire, SPIONs, Co-ferrite, microfluidic system

## Abstract

The detection of magnetic nanoparticles in a liquid medium and the quantification of their concentration have the potential to improve the efficiency of several relevant applications in different fields, including medicine, environmental remediation, and mechanical engineering. To this end, sensors based on the magneto-impedance effect have attracted much attention due to their high sensitivity to the stray magnetic field generated by magnetic nanoparticles, their simple fabrication process, and their relatively low cost. To improve the sensitivity of these sensors, a multidisciplinary approach is required to study a wide range of soft magnetic materials as sensing elements and to customize the magnetic properties of nanoparticles. The combination of magneto-impedance sensors with ad hoc microfluidic systems favors the design of integrated portable devices with high specificity towards magnetic ferrofluids, allowing the use of very small sample volumes and making measurements faster and more reliable. In this work, a magneto-impedance sensor based on an amorphous Fe_73.5_Nb_3_Cu_1_Si_13.5_B_9_ wire as the sensing element is integrated into a customized millifluidic chip. The sensor detects the presence of magnetic nanoparticles in the ferrofluid and distinguishes the different stray fields generated by single-domain superparamagnetic iron oxide nanoparticles or magnetically blocked Co-ferrite nanoparticles.

## 1. Introduction

Magnetic nanoparticles (MNPs) suspended in aqueous or non-aqueous liquids (ferrofluids) have the potential to improve the efficiency of relevant applications in key areas of society, including medicine, environmental treatment, and mechanical engineering [1,2,3,4].

In the field of water purification, MNPs have emerged as a promising material due to their large surface area and fast response to magnetic fields [5,6,7,8,9]. The former enables the effective trapping of large amounts of contaminants through adsorption or catalytic degradation, while the latter facilitates their separation from the treated water. However, MNPs themselves can act as contaminants and are potentially harmful to the environment and human health [6,10]. Therefore, a reliable assessment to determine the complete removal of MNPs from water resources (after their use for contaminant removal) is a significant undertaking that can be addressed with the development of dedicated sensors.

Contextually, in the field of biomedicine, the detection of the stray magnetic field generated by MNPs with selected surface functionalization promotes the development of a range of diagnostic sensors and lab-on-chip devices with increasing sensitivity, biocompatibility, reliability, safety, and energy efficiency [2,11,12,13,14,15,16]. MNP-based biosensors make biomedical diagnostics fast, simple, robust, and high-throughput, improving the detection, separation, and transport of various bioanalytes [14,17], such as cancer biomarkers, which are present at very low concentrations in the early stages of the disease [18].

In both aforementioned fields, the crucial goal of detecting MNPs and quantifying their concentration in a liquid medium is leading to the development of a large number of magnetic sensors [19,20,21,22], among which those based on the magneto-impedance effect are of great interest due to their exceptional sensitivity to the small magnetic fields (up to 10^−12^ T [23]), simple fabrication process, and relatively low cost [24,25,26,27,28].

In particular, the magneto-impedance (MI) effect is defined as a significant change in the electrical impedance of a soft ferromagnetic material when an alternating current flows through it and an external magnetic field is present [29,30,31,32,33].

Improving the sensitivity of MI sensors to the stray magnetic field generated by the MNPs is an interesting ongoing multidisciplinary research topic [16,25,26,28]. This goal is being pursued by exploring a wide range of soft magnetic materials as sensing elements with different compositions, microstructures, and shapes, including microwires, films, and ribbons [34,35,36,37,38]. Furthermore, with equal importance, attention is being paid to customizing the magnetic properties of MNPs by varying their composition, shape, size, functionalization, and concentration in the ferrofluid [25,35,36,37,38].

The combined application of MI sensors for the detection of MNPs suspended in a liquid medium with a microfluidic system offers promising opportunities for the design of relatively inexpensive miniaturized integrated portable devices with high specificity and sensitivity [16,17,39]. In particular, the milli- and microfluidic chips enable the use of very small volumes of liquid, reducing the amount of sample required and the amount of waste produced, as well as making measurements faster and more reliable [24,40].

This study presents a magneto-impedance (MI) sensor designed to detect the stray magnetic field generated by a ferrofluid. The sensor utilizes amorphous Fe_73.5_Nb_3_Cu_1_Si_13.5_B_9_ microwire as the MI sensing element, positioned within a custom millifluidic chip that facilitates the flow of the ferrofluid around the wire.

The response of the MI sensor is tested using two ferrofluids with different magnetic behaviors. The first (i.e., the commercial Synomag ferrofluid) contains single-domain superparamagnetic iron-oxide nanoparticles (SPIONs), which are characterized by anhysteretic behavior and zero magnetic moment in the absence of an applied external magnetic field; the second contains magnetically blocked Co-ferrite nanoparticles, which exhibit a net magnetic moment at zero applied magnetic field. The Co-ferrite nanoparticles are synthesized by a co-precipitation method, and before incorporating them into the liquid medium, their structural and morphological properties as well as the magnetic behavior are studied in detail.

## 2. Materials and Methods

### 2.1. Materials

Co-ferrite nanoparticles were synthesized using the conventional co-precipitation method described in [41]. In brief, an aqueous solution of cobalt(II) nitrate hexahydrate and iron(III) chloride hexahydrate with a 1:2 molar ratio was prepared. The pH of the solution was raised to 13 by adding 10 M aqueous NaOH, and then the solution was heated to 100 °C under nitrogen flow and stirring. After 30 min, iron(III) nitrate nonahydrate was added to the solution to achieve a Co^2+^/Fe^3+^ molar ratio of 1:3, followed by cooling to room temperature. The precipitate was decanted using a magnet and washed multiple times with deionized water until reaching a neutral pH. The nanoparticles are dispersed in aqueous medium, resulting in a stable ferrofluid with selected concentrations *c* equal to 0.8, 4.0, 8.1, 16.2, and 28.3 mg/mL.

Ferrofluid containing superparamagnetic iron-oxide nanoparticles (SPIONs) was purchased from Micromod Partikeltechnologie GmbH, Rostock, Germany [42]. The selected concentrations of SPIONs in the liquid medium are *c* = 0.76, 1.4, 7.6 mg/mL. The SPIONs are composed of iron oxide (mainly γ-Fe_2_O_3_) and result in a multicore structure with a nanoflower shape covered by a dextran shell. The magnetic core size is 9.0 ± 1.7 nm, while the particle size is ≈66 nm [42].

The amorphous magnetic wire of nominal composition Fe_73.5_Nb_3_Cu_1_Si_13.5_B_9_ (FINEMET) was produced by the melt spinning in water technique [43,44]; it is called Fe-based wire in the following text.

### 2.2. Characterization Techniques

The structure and crystallinity of Co-ferrite NPs were investigated by X-ray diffraction (XRD) in a Panalytical X’Pert PRO MPD diffractometer with Cu K_*α*_ radiation. The dried nanopowders were placed in the cavity of a silicon zero-background sample holder and analyzed in Bragg–Brentano configuration. The atomic ratio between Fe and Co in Co-ferrite NPs was estimated by standardless semi-quantitative analysis through energy dispersive spectrometry coupled to a scanning electron microscope (SEM-EDS) after deposition of the nanoparticles on the carbon adhesive tape used for electron microscopy.

Morphology of the Co-ferrite NPs was determined by transmission electron microscopy (TEM, JEM-2100, JEOL, Tokyo, Japan). TEM images were analyzed by the open-source software ImageJ [45] to estimate the NP size distribution.

Magnetic characterization of all samples was performed at room temperature with a highly sensitive vibrating sample magnetometer (VSM, LakeShore, Carson, CA, USA) operating in the magnetic field range of ±17 kOe. Hysteresis loops of the magnetic wire were evaluated by applying the magnetic field along both the longitudinal (i.e., parallel, PA) and transverse (i.e., perpendicular, PE) directions to its major geometric axis. In contrast, SPIONs and Co-ferrite nanoparticles in powder form were randomly dispersed in a suitable sample holder whose diamagnetic signal was duly taken into account and properly subtracted.

Moreover, isothermal residual magnetization (IRM) and continuous demagnetization remanence (DCD) curves were also measured [46] for Co-ferrite nanoparticles. The IRM curve reports the remanence values obtained from a magnetic field progressively increasing towards positive values applied to the initially demagnetized magnetic sample. Conversely, the DCD curve reports the remanence values obtained from a magnetic field progressively increasing towards negative values applied to the magnetic sample initially at positive remanence [46].

**Figure 1:**
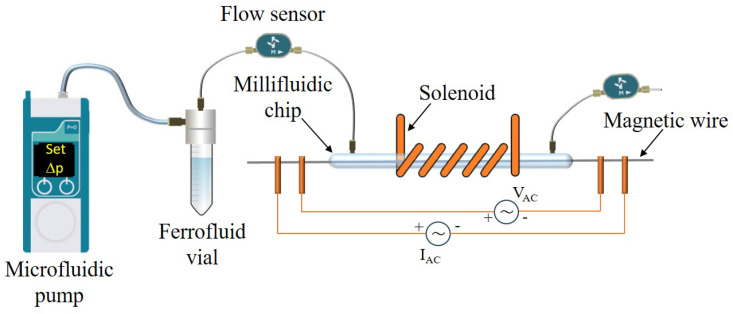
Scheme of the microfluidic system.

A microfluidic system was assembled with the objective of facilitating the flow of the ferrofluid around the Fe-based wire. A schematic representation of this system is provided in Figure 1.

The millifluidic chip consists of a commercial quartz channel with a length of 13 cm and an inner/outer diameter of 1/1.5 mm. The quartz channel ends were inserted into plastic nozzles, which were threaded by lathe to connect the chip to a vial containing the magnetic ferrofluid via external fluorinated ethylene-propylene (FEP) tubes with an inner/outer diameter of 0.5/1.58 mm.

The magnetic wire was positioned within the quartz tube, with the two ends passing through the chip to create a dry area available for the electrical contacts used to measure the magneto-impedance effect. The application of glue ensured that the system was perfectly watertight.

The ferrofluid was made to flow into the chip via an advanced pressure-based flow control system (LineUp Flow EZ, Fluident). In the microfluidic circuit, two flow sensors were positioned at the ends of the chip to measure the flow rate and thus facilitate a reliable estimate of the velocity of the ferrofluid inside the quartz tube as a function of the pressure set on the flow control. When the microfluidic system was turned on, the chip was filled with ferrofluid, which completely submerged the magnetic wire. The entire chip was placed inside a solenoid, which was used to apply the DC magnetic field along the longitudinal axis of the magnetic wire. Some photos of the experimental microfluidic setup, millifluidic chip, and section of the quartz tube are available in the Supplementary Materials; in this paper, the term microfluidic refers to the entire experimental system used, as the pump is suitable for microliter volumes and the FEP tubes are in the micrometer range, while the term millifluidic refers only to the chip used due to the size of its inner diameter.

Magneto-impedance measurements were performed on the Fe-based wire using a conventional four-contact volt-ampere technique in the presence of an external DC field.

The four electrical contacts were made using conductive silver paste on the wire ends outside the millifluidic chip. The two external contacts were used to drive the sinusoidal AC drive current ($I_{AC}$) through the wire, while the two internal ones were used to measure the voltage signal ($V_{AC}$) across the wire, see Figure 1.

A signal generator (SD6022X Siglent, Helmond, The Netherlands) was exploited to generate $I_{AC}$, the frequency of which was set in the range 0.5–1.5 MHz, while the intensity was estimated to be 20 mA peak-to-peak by measuring the voltage across a precisely known resistor placed in series with the magnetic wire. A digital multimeter (3478A, Hewlett Packard, Palo Alto, CA, USA) was used to measure the voltage signal across the Fe-based magnetic wire (whose resistance was 26 Ω). The impedance of the wire is given by *Z* = $V_{AC}/I_{AC}$.

The impedance measurements were performed as a function of the external DC magnetic field (generated by a solenoid powered by a Hewlett Packard 6654A DC power supply) with an amplitude in the range of *H* = ±150 Oe and directed along the main axis of the magnetic wire. The modulation of the external DC field affected the configuration of the magnetic domains in the wire, leading to a large variation in the measured voltage ($\Delta V_{AC}$) and consequently in the impedance (ΔZ). The magneto-impedance ratio (MI) is defined as

(1) $$\frac{\Delta Z}{Z}\left(\%\right)=\frac{Z\left(H\right)-Z\left(H_{max}\right)}{Z(H_{max})}\times 100$$

where $Z(H_{max})$ is the impedance measured at the maximum amplitude of the applied longitudinal DC field.

## 3. Results and Discussion

### 3.1. Structural and Morphological Characterization

**Figure 2:**
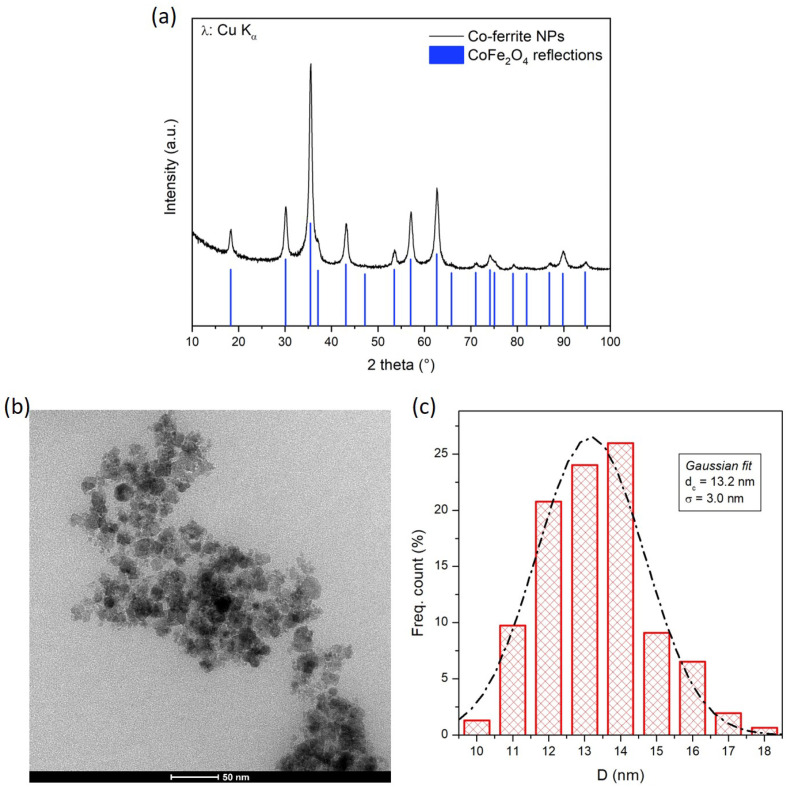
Structural and morphological properties of Co-ferrite NPs: (**a**) XRD pattern, together with the reference lines of CoFe_2_O_4_; (**b**) representative TEM image; (**c**) NP size distribution obtained by the statistical analysis of TEM images and fitted by a Gaussian function (dashed line).

The XRD pattern of the Co-ferrite NPs is shown in Figure 2a: the distinctive peaks reveal that the NPs are crystalline materials and all the reflections are compatible with the ones of cubic iron-spinel phases like magnetite and its substituted relatives such as CoFe_2_O_4_, where all the Fe^2+^ ions have been replaced by Co^2+^ ions. Since these two phases have the same cubic structure and very similar lattice parameters, their XRD pattern is almost identical; consequently, distinguishing cobalt ferrite from magnetite on the basis of X-ray diffraction is very difficult. For this reason, the actual presence and concentration of Co ions in the synthesized NPs was checked and confirmed by SEM-EDS elemental semi-quantitative analysis: the atomic ratio between Co and Fe turned out to be 1:3, corresponding to the one used in the synthesis recipe. Thus, the actual composition of the Co-ferrite NPs can be written as Co_0.75_Fe_2.25_O_4_. The crystallite size of the Co-ferrite NPs, calculated from XRD by means of Scherrer’s formula after subtraction of the instrumental contribution to peak broadening, is around 11 nm.

A representative TEM image of the Co-ferrite NPs is shown in Figure 2b. The NPs appear well defined, with an almost spherical shape. Statistical analysis of some TEM images yields the NP size distribution shown in Figure 2c, which is well fitted by a Gaussian function with a mean value of 13.2 nm and a standard deviation of 3.0 nm, in reasonable agreement with the value obtained by XRD analysis.

**Figure 3:**
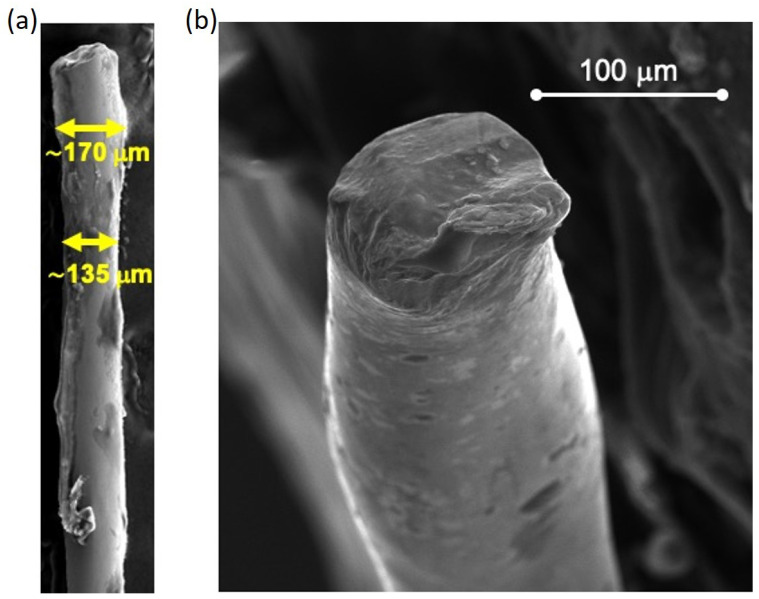
SEM images of the Fe-based wire: (**a**) top view and (**b**) cross section.

The morphology of the Fe-based wire is investigated by the SEM images shown in Figure 3. The top-view image (panel a) reveals that the diameter of the wire is not perfectly constant but is characterized by fluctuations of a few tens of microns around an average value of 150 μm. Furthermore, the cross-sectional image (panel b) shows a homogeneous morphology of the wire throughout its thickness with no visible cracks, crystals, or inclusions.

### 3.2. Magnetic Characterization of the MNPs

**Figure 4:**
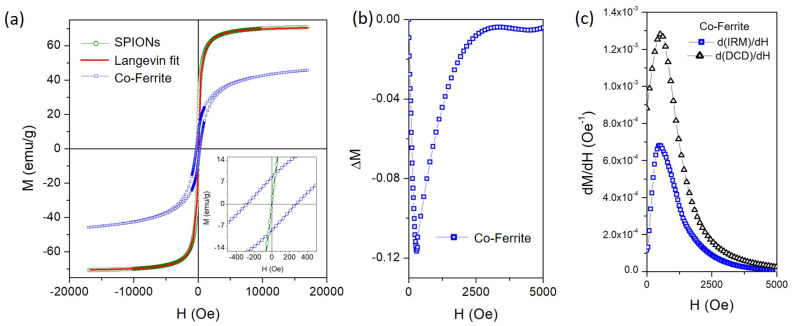
(**a**) Room-temperature hysteresis loop for Co-NPs (blue curve) and SPIONs (green curve) fitted with a Langevin curve (red line); (**b**) ΔM(*H*) curve obtained combining linearly the IRM and DCD measurements for the Co-NPs (see text for details); (**c**) field derivative for IRM and DCD curves for the Co-NPs.

Room-temperature hysteresis loops for SPIONs and Co-NP samples in dry powder form are reported in Figure 4a.

The magnetization curve for SPIONs shows an anhysteretic behavior with a sigmoidal trend with a non-saturating behavior at high fields. This curve is well fitted by the superimposition of two Langevin curves (red line), proving the genuine superparamagnetic state of these nanoparticles [47,48].

The value of the saturation magnetization ($M_{S}$) is estimated from the high-field extrapolation of the Langevin fit curve, which gives $M_{S}\approx$ 71 emu/g, a value lower than the saturation magnetization of bulk magnetite (92 emu/g) [49] due to the non-negligible role of magnetic disorder induced by surface magnetic anisotropy. Moreover, Langevin’s fit leads to estimate the average size of SPIONs at around 12 nm, in good agreement with the size values obtained from TEM and XRD analyses.

Conversely, the M(H) curve for the Co-NP sample shows a hysteretic behavior with a sigmoidal trend, characterized by an unsaturated behavior at the maximum applied field. The $M_{S}$ values are estimated by fitting the high-field portion of the M(H) curve with the well-known expression [50] $M\left(H\right)=M_{S}\left(1-\delta /H-\gamma /H^{2}\right)+\chi H$, where δ and γ are free parameters, while χ is set to zero to neglect any paramagnetic contribution. This procedure gives $M_{S}\approx$ 50 emu/g; such a value is fully compatible with Co-ferrite nanoparticles [51,52], although it is lower than that of bulk Co-ferrite (80 emu/g) [49]. This reduction can be attributed to the effects of a non-equilibrium distribution of Co^2+^ and Fe^2+/3+^ cations in the spinel ferrite structure [51] and spin canting/disorder on the nanoparticles surface [51]. Additionally, the M(H) curve is characterized by a coercive field ($H_{C}$) value of about 280 Oe and a remanence magnetization ($M_{R}$) of about 8.5 emu/g (see the inset of Figure 4a).

The remanence curves, i.e., isothermal remanence magnetization (IRM) and dc-demag- netization remanence (DCD), for the hysteretic Co-NP sample are measured by means of VSM and are reported in the Supplementary Materials.

These curves represent non-equilibrium magnetic states measured after the application and removal of a positive DC field with increasing amplitude on the sample in different initial magnetic configurations: demagnetized state and saturation remanence for IRM and DCD measurements, respectively [46].

The parameters of isothermal remanence coercivity ($H_{C_{IRM}}$) and demagnetization remanence coercivity ($H_{C_{DCD}}$) indicate the field at which the IRM curve is equal to 0.5 and the field at which the DCD curve crosses zero, respectively; the estimated values for Co-NPs are $H_{C_{IRM}}$ = 927 Oe and $H_{C_{DCD}}$ = 850 Oe.

The evidence of $H_{C_{IRM}}>H_{C_{DCD}}$ indicates that the Co-NP sample turns out to be more difficult to magnetize than to demagnetize; consequently, it can be inferred that interactions among Co-ferrite NPs occur and play a non-negligible role in the magnetization process [46,53,54]. This assumption is also supported by the shape of the ΔM(H) curve (see Figure 4b) obtained by combining linearly the IRM and DCD measures: ΔM=DCD(H)−[1−2IRM(H)]. In particular, the well-defined negative dip confirms the existence of dipole–dipole inter-particle interactions, which tends to demagnetize the entire sample [55,56,57].

Moreover, the ratios $H_{C_{DCD}}$/$H_{C}$ = 3 and $M_{R}$/$M_{S}$ = 0.17 collocate the studied Co-NPs in the pseudo-single-domain (PSD) region of the Day’s plot [58].

The field derivative for both normalized remanence curves is reported in Figure 4c to highlight the switching field distribution required to magnetize the Co-NP sample. Both curves show a rapid increase from a low value to a well-defined maximum. The presence of a single peak indicates that a single reversal mechanism in the magnetization process takes place [55]. After that, the curves very slowly decrease, approaching zero, to a field value of approximately *H* = 5 kOe, which indicates the minimum amplitude of the magnetic field required to activate all irreversible mechanisms of the magnetization process. At this value, the hysteresis curve shows the overlap of the two branches (see Figure 4a) but has not yet reached its maximum value, which will only be achieved by increasing the applied magnetic field by a rotational and reversible mechanism of magnetization.

### 3.3. Magnetic Characterization of Magnetic Fe-Based Wire

**Figure 5:**
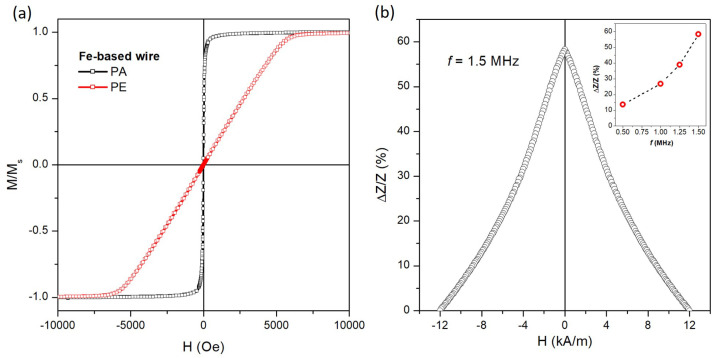
(**a**) Room-temperature hysteresis loops of the Fe-based wire evaluated along the directions parallel (PA) and perpendicular (PE) to its geometric axis; (**b**) field dependence of ΔZ/ZV at *f* = 1.5 MHz for the Fe-based wire and the frequency dependence of the ΔZ/ZV at zero applied field.

Room-temperature hysteresis loops for the Fe-based wire obtained by applying a magnetic field along the directions parallel (PA) and perpendicular (PE) to its geometric axis are reported in Figure 5a; both curves are normalized to the value of the magnetic moment at *H* = 10 kOe.

The M(H) curve along PA (black symbols) shows a magnetization reversal in a narrow magnetic field range by a sharp and irreversible jump of the magnetic moment, defining a high magnetic susceptibility at the coercive field ($\chi_{Hc}$ = 1.64 × 10^−2^ Oe^−1^) and magnetic remanence ($M_{r}/M_{s}$ = 0.75), as well as an extremely low coercive field (below the VSM resolution). Conversely, the magnetization reversal along the PE direction (red symbols) occurs by a continuous rotation of the magnetic vector over a wide field range (*H* = 6.5 kOe) with a perfectly linear behavior, resulting in a low magnetic susceptibility ($\chi_{H_{c}}$ = 1.85 × 10^−4^ Oe^−1^) and a magnetic remanence and coercive field close to zero. No irreversible processes characterized by magnetization jumps are visible.

These soft magnetic properties of the Fe-based wire are consistent with an ideal single-domain structure characterized by an extremely low crystalline anisotropy with the easy and hard axes aligned along the PA and PE directions, respectively. Such effective magnetic anisotropy is mainly dominated by the contribution of the shape anisotropy resulting from the high aspect ratio of the wire [49].

An example of the magneto-impedance (MI) effect on the magnetic Fe-based wire is shown in Figure 5b, where the ΔZ/Z ratio is reported as a function of the DC magnetic field applied along the PA direction of the wire. The frequency of the AC current applied to the wire is 1.5 MHz.

The ΔZ/ZV curve shows a single peak at *H* ≈ 0 Oe and a monotonous and symmetric decrease as the magnetic field amplitude increases.

Such behavior of the MI curve indicates that the easy magnetization direction for magnetic anisotropy is nearly parallel to the wire axis and the transversal magnetization is always dominated by the rotational process, in perfect agreement with the evidence obtained from the hysteresis loops (see Figure 5a) [29,33,59].

The dependence of the maximum of ΔZ/Z (*H* = 0) as a function of the AC current frequency is shown in the inset of Figure 5b. The observed increase is due to the relationship between the current frequency and the skin penetration depth. In fact, as the frequency increases, the skin penetration depth decreases, and thus, the circular permeability increases, leading to an increase in *Z* [60]. The highest value results at 1.5 MHz, which is the value selected as the working frequency for the detection of MNPs using an amorphous wire magneto-impedance sensor in a microfluidic setup.

### 3.4. Microfluidic Detection of MNPs Using Amorphous Wire Magneto-Impedance Sensor

A magneto-impedance signal on Fe-based wire can be exploited to detect the presence of a magnetic ferrofluid surrounding it, allowing the wire to act as a sensing element. The ferrofluid is pumped by the microfluidic system up to surround the Fe-based wire, after which it is stopped before measuring the magnetic impedance.

**Figure 6:**
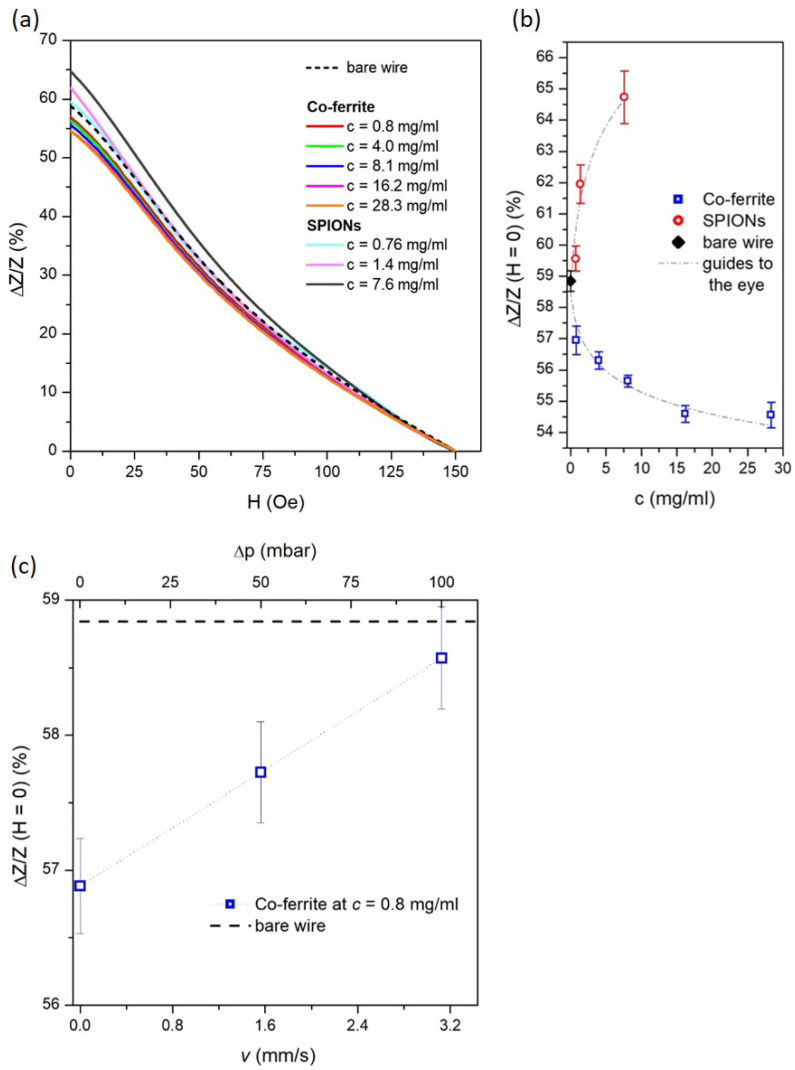
(**a**) Field dependence of ΔZ/ZV at *f* = 1.5 MHz for the Fe-based wire immersed on the static ferrofluid at different concentrations of Co-ferrite NPs or SPIONs; (**b**) ΔZ/ZV values at zero DC field as a function of the static ferrofluid concentration for Co-ferrite NPs and SPIONs; (**c**) ΔZ/ZV(*H* = 0) ratio as a function of the pressure difference (Δp) set to the flow of the Co-ferrite ferrofluid.

Figure 6a shows that the ΔZ/Z (%) curve of the bare wire (here displayed as a dashed line and already shown previously in Figure 5b) is significantly modified by the presence of SPION and Co-ferrite NPs dispersed in a liquid medium at selected concentrations; each curve shown in Figure 6a is the average of six repeated measurements under the same experimental conditions.

In particular, the magnitude of the MI effect is adjusted according to the type and concentration of the NPs; conversely, the shape of the ΔZ/Z (%) curve maintains its maximum at *H* = 0 Oe with a monotonous trend decreasing as the external magnetic field increases. This evidence shows that the ferrofluid magnetically interacts with the wire but does not affect its uniaxial magnetic anisotropy. In fact, the magnetic NPs generate a stray field that affects the MI behavior by superimposing on the external longitudinal DC field and the transverse AC magnetic field generated by the AC excitation current.

The ΔZ/Z values at *H* = 0, corresponding to the maximum MI variation, are shown as a function of nanoparticle concentration in Figure 6b. The SPIONs and Co-ferrite NPs affect the MI signal in opposite ways with increasing concentration. The former leads to an increase in the MI amplitude, while the latter induces a reduction. The explanation for this opposite behavior should be sought in the different magnetic states of the two types of NPs [35,36,37,38,61].

As a matter of fact, the magnetic behavior of the SPIONs, which refers to a single-domain magnetic state with extremely low magnetic anisotropy energy and negligible magnetic interaction (see Figure 4a and Section 3.2 for more details), allows the SPIONs themselves to instantaneously generate a magnetic moment in response to the transverse AC and longitudinal DC magnetic fields applied during magneto-impedance measurements. Therefore, under the action of the AC transverse magnetic field alone (i.e., *H* = 0), whose magnitude is related to the current intensity and whose maximum is near the wire surface, the magnetic moment of SPIONs quickly reacts, promoting the domain wall mobility of the Fe-based wire, thus enhancing the MI effect as a function of SPION concentration, as shown in Figure 6b [37,38]. The superimposition of the longitudinal DC magnetic field progressively overcomes the effect of the transverse field and (depending on its strength) gradually orients the magnetic moments of the SPIONs along the major axis of the wire. This results in a reduction in the transverse magnetic permeability of the wire, resulting in a monotonic reduction of the ΔZ/Z(%) curve as a function of *H*, as shown in Figure 6a.

In contrast, the hysteretic magnetic behavior of the Co-ferrite nanoparticles, characterized by a significant magnetic anisotropy energy even in the absence of an applied magnetic field (see Figure 4a and Section 3.2 for more details), hinders the action of the transverse AC magnetic field, which is no longer sufficient to orient the magnetic moments of the Co-ferrite nanoparticles in a transverse direction.

Furthermore, the intrinsic magnetic moments of these nanoparticles favor dipole magnetic interactions, which end up with the formation of nanoparticle arrangements such as clusters or chains. The resulting magnetic stray field tends to hinder the domain wall motion in the Fe-based wire, leading to a progressive reduction of the MI effect as a function of the Co-NP concentrations, as shown in Figure 6b [35,36,61]. Again, the longitudinal DC magnetic field gradually orients the magnetic moments of Co-ferrite NPs or clusters along the major axis of the wire; thus, a monotonic reduction of the ΔZ/Z(%) curve as a function of *H* is measured, as shown in Figure 6a.

The evolution of ΔZ/Z (*H* = 0) values as a function of *c* was fitted to determine the calibration curve of the amorphous wire MI sensors for both types of nanoparticles.

In particular, the same logarithmic function, represented by the equation ΔZ/Z (*H* = 0) = $P_{1}+P_{2}\times \ln\left(c+P_{3}\right)$, was able to fit well the experimental data obtained from both nanoparticle systems with suitable coefficients ($P_{1}$, $P_{2}$, and $P_{3}$), as shown by the dashed gray lines in Figure 6b.

Moreover, these calibration curves enable the determination of the limit of detection (LOD) of the studied MI sensor, which is a pivotal parameter for characterizing it [62]. The LOD is defined as the smallest concentration of nanoparticles that produces a statistically significantly greater MI signal than that obtained from the repeated measurements of the bare wire [62,63]. This quantity is given by LOD = 3.3 $\sigma_{b}$/*m*, where $\sigma_{b}$ is the standard deviation of the MI value for the bare wire and *m* is the slope of the calibration curve for c → 0 [63]. The LOD estimates are ≈0.3 and 0.7 mg/mL for SPIONs and Co-ferrite, respectively.

In addition, the detection range of the MI sensor is evaluated using Co-ferrite ferrofluid, taking advantage of the availability of more concentrated samples. It is approximately between 0 and 16.2 mg/ml; in fact, at higher concentrations, the calibration curve becomes so flat that the measured MI values tend to be statistically indistinguishable. It is evident that the experimental ΔZ/Z (*H* = 0) value 54.55 ± 0.41 % obtained for *c* = 28.3 mg/mL overlaps with the value 54.59 ± 0.27 % obtained for *c* = 16.2 mg/mL. A similar detection range would also be expected for the SPION ferrofluid if the calibration curve were extended to higher concentrations.

Magneto-impedance measurements are also capable of detecting magnetic nanoparticles under continuous flow of the ferrofluid.

The trend of ΔZ/Z (*H* = 0) as a function of the velocity (*v*) of the Co-ferrite ferrofluid at *c* = 0.8 mg/ml through the millifluidic chip is shown in Figure 6c. The flow velocity is related to the pressure difference (Δp) set in the pressure-based flow control system and is determined by averaging the flow rate measurements through two flow sensors placed in the microfluidic setup (see Figure 1).

In the range Δp = 0–100 mbar, the ferrofluid flows inside the millifluidic chip with a *v* = 0–3.125 mm/s dominated by a laminar flow as confirmed by the Reynolds number (Re) of 3.125 for maximum speed. The Reynolds number is calculated by Re=ρvs.L/μ, where *v* is the flow velocity, *L* = 1 mm is the inner diameter of the quartz channel, and ρ = 1000 kg/m^3^ and μ = 0.001 Pa·s are the density and viscosity of the ferrofluid, respectively, which are reasonably approximated to those of pure water (at room temperature) due to the very low concentration of nanoparticles.

As a matter of fact, a linear approach of the ΔZ/Z (*H* = 0) ratio from the value measured in static flow conditions (*v* = 0) towards the value of the bare wire is observed with increasing *v*.

This behavior can be reasonably explained by considering the competing Brownian and laminar forces that drive the displacement of the Co-ferrite NPs within the millifluidic chip. The balance of these hydrodynamic forces determines the time taken for the NPs to move in the multichannel during MI measurements and thus the efficiency of their magnetic interaction with the Fe-based wire. MI measurements take approximately 2 min due to the time required to bring the DC field to its maximum amplitude.

In the static flow condition (*v* = 0), Brownian forces are dominant, resulting in the random displacement of each NP within the liquid. This results in a high probability of the NPs remaining within the millifluidic chip throughout the duration of the MI measurement. Consequently, the NPs, subjected to AC and DC fields, maximize the interaction with the domain walls of the Fe-based magnetic wire, thereby inducing a substantial variation in the MI signal.

In the continuous flow condition, the hydrodynamic forces typical of the laminar state transport the NPs into the flow. Therefore, the travel time of the Co-ferrite NPs in the millichannel is progressively reduced as the ferrofluid velocity increases. Consequently, during MI measurements, the magnetic interaction of the NPs with the walls of the magnetic wires becomes progressively less effective and the variation in the MI signal becomes less significant.

## 4. Conclusions

A magneto-impedance sensor aimed at detecting the stray magnetic field generated by magnetic nanoparticles dispersed in a ferrofluid was successfully obtained by exploiting an amorphous Fe_73.5_Nb_3_Cu_1_Si_13.5_B_9_ magnetic wire (average diameter of 150 μm), characterized by soft magnetic properties and anisotropy that is easy-axis-aligned along its main geometrical axis.

The detection of the magnetic nanoparticles takes place in a custom millifluidic chip to facilitate the flow of the ferrofluid around the Fe-based wire and to reduce the required sample and the amount of waste produced. In addition, this combined application of MI sensors with a microfluidic system promotes the development of miniaturized and portable devices, making measurements faster and more reliable.

In particular, it is observed that the magnitude of the MI signal is significantly influenced by the intrinsic magnetic properties of the NPs and their concentration in the ferrofluid. The superparamagnetic state of the SPIONs enables them to respond rapidly to the AC transverse magnetic field with a magnetic moment that generates a stray field capable of increasing the transverse magnetic permeability of the wire with a consequent enhancement of the MI effect. Conversely, the magnetically blocked state of the Co-ferrite NPs is characterized by an intrinsic magnetic moment that favors dipole magnetic interaction, resulting in a magnetic stray field that tends to hinder the domain wall motion in the Fe-based microwire. This leads to a reduction in the MI effect.

Under static flow conditions, the MI sensor demonstrates sensitivity to ferrofluid concentration with a detection limit of approximately 0.3 and 0.7 mg/ml for SPION and Co-ferrite, respectively. In dynamic flow conditions, the travel time of the Co-ferrite NPs decreases as a function of fluid velocity, and therefore, the magnetic interaction of the NPs with the wires becomes progressively less effective and the variation of the MI signal is observed to be less significant.

In conclusion, a simple and robust amorphous wire magneto-impedance sensor was proposed that is characterized by a simple fabrication process and relatively low cost. Its sensitivity to the stray magnetic field generated by MNPs in the ferrofluid makes it useful for reliably assessing the complete removal of NPs from water resources (after their use for contaminant removal), as well as for promoting the development of diagnostic sensors and lab-on-chip devices based on the detection of functionalized NPs.

## Data Availability

The data are available from the corresponding author upon reasonable request.

## Supplementary Materials

The following supporting information can be downloaded at: https://www.mdpi.com/article/10.3390/s24154902/s1, - Photo of experimental microfluidic detection setup; - photo of millifluidic chip equipped with nozzles and magnetic wire; - photo of the section of quartz tube; - IRM and DCD curves for hysteretic Co-ferrite NP sample.
