# Supplementary material for: Microfluidic Detection of SPIONs and Co-Ferrite Ferrofluid Using Amorphous Wire Magneto-Impedance Sensor
Source: Sensors (Basel). 2024 Jul 28;24(15):4902. doi: 10.3390/s24154902 (PMC11315026; doi:10.3390/s24154902)
Supplement: Supplementary file 1 [file sensors-24-04902-s001.zip › sensors-3067627-supplementary.pdf]

Supplementary Material

Microfluidic Detection of SPIONs and Co-ferrite Ferrofluid Using Amorphous Wire Magneto-Impedance Sensor

Gabriele Barrera[1],* , Federica Celegato[1], Marta Vassallo[1], Daniele Martella[2,3], Marco Coïsson[1], Elena S. Olivetti[1], Luca Martino[1], Hüseyin Sözeri[4], Alessandra Manzin[1] and Paola Tiberto[1]

[1] Department of Advanced Materials Metrology and Life Science, Istituto Nazionale di Ricerca Metrologica (INRiM), Strada delle Cacce, 91, 10135 Torino, Italy.

[2] European Laboratory for Non Linear Spectroscopy (LENS), via N. Carrara, 50019, Sesto Fiorentino, Firenze, Italy.

[3] Department of Chemistry "Ugo Schiff", University of Florence, Via N.Carrara, 3-13, 50019, Sesto Fiorentino, Firenze, Italy.

[4] Magnetics Laboratory, TÜBITAK Ulusal Metroloji Enstitüsü (UME), Gebze Yerle ̦skesi, 41470 Kocaeli, Turkey.

* Correspondence: g.barrera@inrim.it

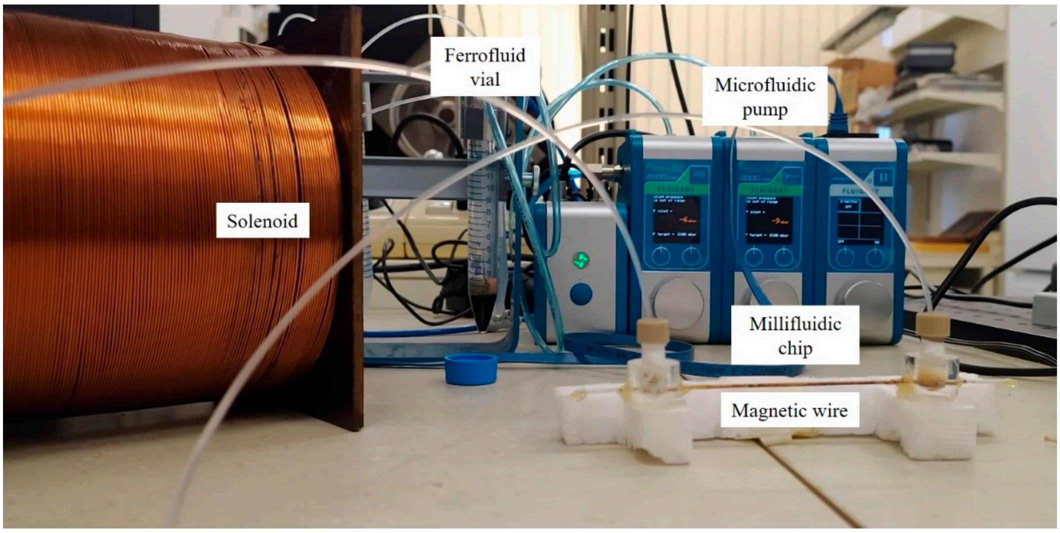

Photo of experimental microfluidic detection setup.

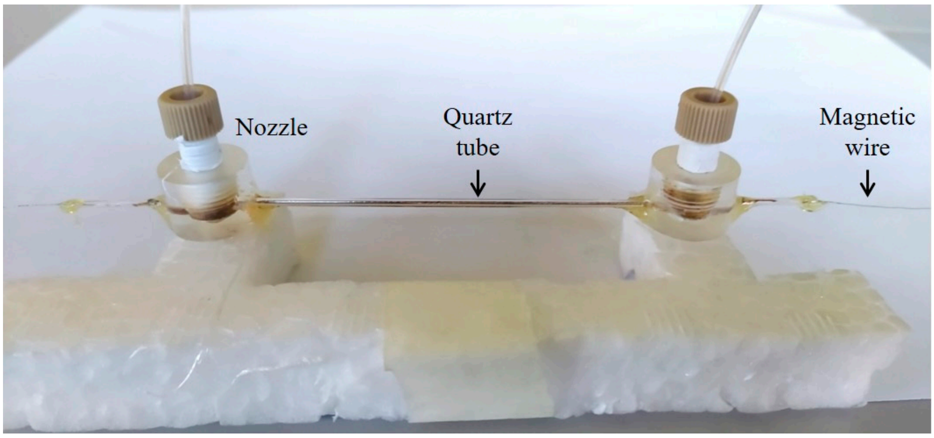

Photo of millifluidic chip equipped with nozzles and magnetic wire.

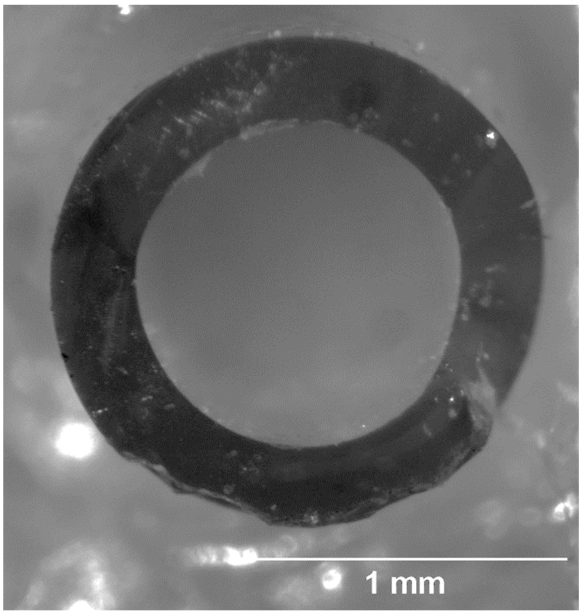

Photo of the section of quartz tube. The inner diameter is 1 mm.

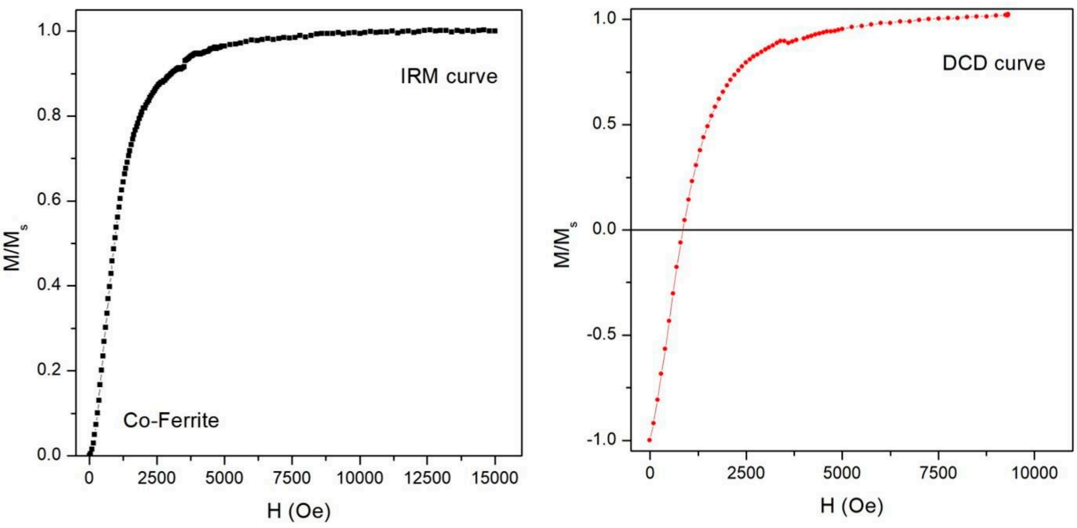

Isothermal remanence magnetization (IRM) and DC-demagnetization remanence (DCD) curves for the hysteretic Co-ferrite NP sample.
